# Untargeted Ultrahigh-Performance Liquid Chromatography-Hybrid Quadrupole-Orbitrap Mass Spectrometry (UHPLC-HRMS) Metabolomics Reveals Propolis Markers of Greek and Chinese Origin

**DOI:** 10.3390/molecules26020456

**Published:** 2021-01-16

**Authors:** Maria-Ioanna Stavropoulou, Aikaterini Termentzi, Konstantinos M. Kasiotis, Antigoni Cheilari, Konstantina Stathopoulou, Kyriaki Machera, Nektarios Aligiannis

**Affiliations:** 1 Department of Pharmacognosy and Natural Products Chemistry, Faculty of Pharmacy, National and Kapodistrian University of Athens, Panepistimiopolis Zografou, 11527 Athens, Greece; mstavropoul@yahoo.gr (M.-I.S.); cheilarianti@pharm.uoa.gr (A.C.); kstatho@pharm.uoa.gr (K.S.); 2 Laboratory of Pesticides’ Toxicology, Department of Pesticides Control and Phytopharmacy, Benaki Phytopathological Institute, 8 St. Delta Street, Kifissia, 14561 Athens, Greece; a.termentzi@gmail.com (A.T.); K.Kasiotis@bpi.gr (K.M.K.); k.machera@bpi.gr (K.M.)

**Keywords:** Mediterranean propolis, poplar propolis, Greek propolis, Chinese propolis, UHPLC-HRMS, chemometrics

## Abstract

Chemical composition of propolis depends on the plant source and thus on the geographic and climatic characteristics of the site of collection. The aim of this study was to investigate the chemical profile of Greek and Chinese propolis extracts from different regions and suggest similarities and differences between them. Untargeted ultrahigh-performance liquid chromatography coupled to hybrid quadrupole-Orbitrap mass spectrometry (UHPLC-HRMS) method was developed and 22 and 23 propolis samples from Greece and China, respectively, were analyzed. The experimental data led to the observation that there is considerable variability in terms of quality of the distinctive propolis samples. Partial least squares - discriminant analysis (PLS-DA) and orthogonal partial least squares-discriminant analysis (OPLS-DA) models were constructed and allowed the identification of significant features for sample discrimination, adding relevant information for the identification of class-determining metabolites. Chinese samples overexpressed compounds that are characteristic of the poplar type propolis, whereas Greek samples overexpress the latter and the diterpenes characteristic of the Mediterranean propolis type.

## 1. Introduction

Propolis (bee glue) is a natural resinous product that honeybees collect from several plant leaves, buds, and exudates and mix it with beeswax and salivary enzymes [1]. The word “propolis” derives from Greek words “pro” and “polis” meaning “in front of the city” due to the protective role of propolis for bee colonies against microorganisms, insects, and adverse weather conditions [2,3]. Propolis has been considered of great value for both honeybees and humans. Propolis has been used as a folk medicine for centuries due to its multiple pharmacological properties, including antibacterial, antifungal, antiviral, anti-inflammatory, antiparasitic, antioxidant, hepatoprotective, immunostimulant, antitumor, and cytostatic activity [4]. The chemical composition of propolis varies highly depending on the plant source, the site of collection, the season, and the type of bees. More than 300 constituents have been identified in propolis [5]. In particular, propolis samples from temperate climate zones, such as Europe, North America, and the non-tropical regions of Asia, are mainly composed of the bud exudates of *Populus* species and are rich in flavonoids such as pinocembrin, galangin, and chrysin and in phenolic acids such as caffeic acid, ferulic acid, and cinnamic acid [1]. This type of propolis is known as poplar type propolis. European propolis contains mainly poplar type phenolics [6]. In tropical and subtropical regions, bees have other plant sources, so composition of propolis coming from these areas is highly diverse and totally different from that of the poplar type. In more detail, Brazilian propolis is rich in prenylated derivatives of p-coumaric acid and of acetophenone. Its main plant source proved to be *Baccharis dracunculifolia*. Cuban propolis is rich in polyisoprenylated benzophenones coming from the floral resin of *Clusia rosea* and is distinct from both European and Brazilian types [7]. Mediterranean propolis, on the other hand, is characterized by the presence of diterpene components, such as isocupressic, pimaric, and communic acids, isoagatholal, agathadiol, and totarol, whereas it has almost no phenolics [6,8]. Its botanical origin is yet unidentified, but, especially for the Mediterranean propolis coming from Greece, on the basis of the diterpenic profile, the source plant should be some conifer species (Cupressaceae or Pinaceae) [8].

The aim of this study was to investigate the chemical profile of Greek and Chinese propolis extracts from different regions and suggest similarities and differences between them. Greece, due to geomorphological characteristics, presents great biodiversity with a high percentage of endemic plants. Previous studies showed that Greek propolis samples are different from the typical European poplar type, since they are rich in terpenes and anthraquinones. In more detail, samples coming from mainland Greece contain low amounts of terpenes and high amounts of flavonoids, phenolic acids and their esters and anthraquinones, whereas samples coming mainly from Greek islands are rich in terpenoids, especially diterpenoids [9]. Some conifer species of the Cupressaceae family seem to be its plant source, while the presence of succinyl derivatives leads to the assumption that species of the Pinaceae participate as well [6]. Recently, Kasiotis et al. have reported the presence of rosmarinic acid, 1-docosene, hexadecane, octacosane, hexacosene, and heptacosane for the first time in Greek propolis [10]. On the other hand, China is the largest producer of propolis worldwide. Many researchers have believed until recently that due to China’s climatic diversity, propolis would vary greatly. Wang et al. (2017) found, through a combination of observation of worker bees and phytochemical analysis, that *Populus canadensis* (a hybrid of *Populus nigra* and *Populus deltoids*) is the plant origin of Chinese propolis. Its main components are characteristic of the poplar type propolis, like benzyl- and phenethyl caffeate, chrysin, pinocembrin, galangin, 5-methoxy pinobanksin, and pinobanksin [11].

Different techniques have been applied for the exploration of propolis’ chemical profiling and the classification of propolis samples [12,13]. Such methods involve metabolic profiling, fingerprinting, and metabolomic studies (identification and/or quantitation of metabolites produced by a biological system at a specific point) with the aid of chemometric tools in order to obtain a better view of vast data. Mass spectrometry (MS) and nuclear magnetic resonance (NMR) are the most commonly employed analytical techniques, especially in the case of analysis of complex natural biological samples [14]. Since propolis is a mixture with great variability, precise and rapid methods are needed.

In this study, propolis samples from different regions of Greece and China were collected and investigated through an untargeted UHPLC-HRMS-based metabolomics analysis workflow. Liquid chromatography coupled to mass spectrometry (LC-MS) has proved to be an effective technique in the analysis of complex natural mixtures, such as propolis [10,12,15]. High sensitivity of the MS technique is a great advantage and provides the potential of discovery of new minor components [14,15] in comparison to NMR. Moreover, MS fingerprinting has been used to compare the composition of propolis with those of plant resins and it can also be used for quick classification of propolis samples without detailed identification of individual components [14,16,17].

In continuation of our efforts to study propolis from different territories by means of NMR [9] and LC-MS [10], we presented a mass spectrometry-based omics strategy to discover discriminating compounds. The variance in propolis’ chemical composition led us to collect as many samples as possible covering regions of Greece and China with different vegetation, geomorphological and weather conditions in order to construct reliable models for future chemometric studies. Finally, this research can contribute towards the establishment of standard compounds linked to a particular propolis’ chemical type and facilitate the urge to develop standardized propolis extracts.

## 2. Results and Discussion

### 2.1. LC-MS Data Acquisition and Processing

**Figure 1:**
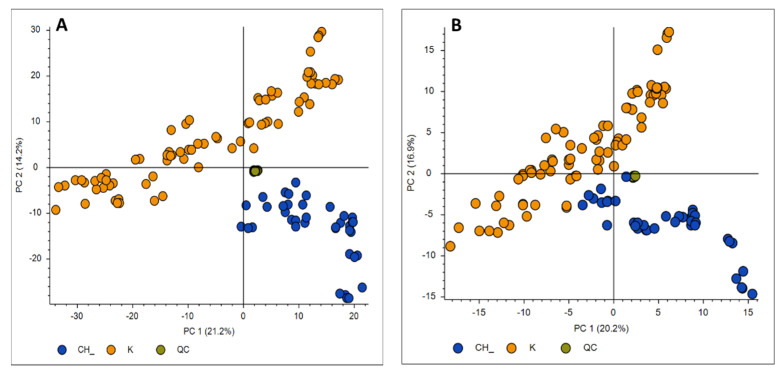
PCA score plots of MS data in the positive (**A**) and negative (**B**) ion modes constructed in the Compound Discoverer software using Pareto scaling for Chinese (CH, blue points) and Greek (K, orange points) propolis samples. QCs (green points) are tightly clustered close in both ion modes.

After the acquisition of propolis samples, the Orbitrap data (.raw files) were entered to the Compound Discoverer software. In order to generate a matrix with the m/z and the retention times of the features and perform a preliminary principal component analysis (PCA), a classical untargeted metabolomics workflow which includes statistics was followed. More specifically, an untargeted metabolomics workflow with the detection of unknown compounds and identification using in-house and online databases was applied. A crucial parameter in the data analysis workflow is the normalization. In metabolomic studies, it is important to reduce any systematic error in instrumental conditions (e.g., drift of the signal intensity of the mass spectrometer), as it influences the accuracy and precision of the analysis, making it difficult to interpret biological data [18,19]. Various data normalization methods were employed in order to minimize instrumental and methodological bias between measurements, so that only biologically relevant differences were present [20,21]. For the Compound Discoverer software, the normalization is based on quality control (QC) area corrections. QC samples were injected periodically, every 6 samples throughout the run to assess instrument stability (Supplementary material, Figure S1). PCA analysis was used in order to explore whether the QC data were closely correlated. Highly variable QC data would mean that the run had failed. QC samples were used in order to normalize the acquired data using the QC-RLSC (QC-based LOESS signal correction) algorithm before the multivariate statistical analysis. For the present study, 50% QC coverage and 30% RSD (relative standard deviation) of the maximum QC area were applied. The normalization type selected was the constant mean (after comparing the results with the constant sum, constant median, and median absolute deviation types). Blank samples were excluded from the procedure. The selection of the normalization algorithm was achieved according to the clustering of QCs. Injections of the QC samples (green points) that are shown to be tightly clustered to the PCA (Figure 1) indicate good instrument performance, thus denoting that the performed analysis is reliable. The tighter a QC data cluster is, the more accurate the obtained data are, given the fact that they are all injected from the same QC sample.

### 2.2. Chemometric Analysis of MS Data

Various multivariate analysis methodologies such as PCA, PLS-DA, and OPLS-DA were employed after the initial data preprocessing. The PCA methodology was performed using Compound Discoverer and SIMCA and was initially used to explore the existence of possible clustering and detect putative outliers, but also to investigate the extent of repeatability of the QC samples in the corresponding analytical runs. Applying PCA, two groups could be easily separated, both in the positive (Figure 1A) and the negative ion modes (Figure 1B): Greek and Chinese samples. The analyses were deemed to be reliable for the downstream statistical evaluation since the QC samples were clustered tightly together in the score plots. The clear separation of clusters in the unsupervised PCA model suggests phytochemical differences between the samples harvested from Greece and China; however, these differences are much more intense in the positive mode as shown in Figure 1.

**Figure 2:**
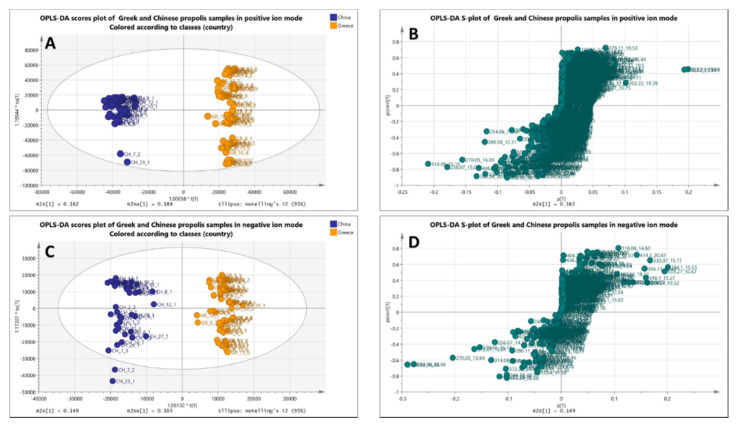
Top: OPLS-DA score plot (**A**) and S-plot (**B**) of Greek (orange) and Chinese (blue) propolis samples (UHPLC-(+)ESI-HRMS). Bottom: OPLS-DA score plot (**C**) and S-plot (**D**) of Greek (orange) and Chinese (blue) propolis samples (UHPLC-(–)ESI-HRMS).

Moreover, data were extracted in Microsoft Excel (.xlsx file format) for further statistical evaluation using the SIMCA software. Supervised methodologies PLS- and OPLS-DA were applied. In such supervised models, the user assigns the samples into predefined groups, which creates the risk of either “artificial”-biased clustering or overfitting. In this case, PLS-DA and OPLS-DA models were validated by elaborating 100 permutation tests showing statistically significant separation between the two groups and the quality of the models was determined by the goodness of fit, goodness of prediction, and cross-validation with R2Y coefficients. Validation data of statistical models can be found in the supporting materials for the positive (PLS-DA, Figure S2, and OPLS-DA, Figure S3) and negative ion modes (PLS-DA, Figure S4, and OPLS-DA, Figure S5). In Figure 2, clear grouping between the Greek and Chinese samples is observed in the negative and positive modes.

### 2.3. Biomarker Investigation

Following the overall chemical profiling of samples, compounds responsible for group clustering were further investigated (Table 1). Variable importance in projection (VIP) and coefficient values extracted from the PLS-DA analysis revealed the most important features in the positive and negative electrospray ionization (ESI) modes (Figure S6) responsible for the differentiation of Greek and Chinese propolis. Moreover, in order to facilitate dereplication approaches, a list of 45 standard compounds (Table S1) that had been reported as propolis components were selected (Benaki Phytopathological Institute in-house chemical library) to generate a standard mixture. High resolution MS/MS spectra were obtained for each full scan ion (Figure S7) in order to perform safe structural elucidation. Structural elucidation of the features responsible for sample clustering was performed by comparison of the chromatographic and spectrometric features of each peak in comparison with data from literature and the standard compounds. The Orbitrap high resolution -MS^2^ profiling was performed in both the positive and negative modes. The high resolving power of 70,000 at the full scan experiments and 35,000 for the MS/MS fragments of the Q-Exactive Orbitrap analyzer in correlation to the accurate mass measurements (Δ m < 1 ppm for both full scan and MS/MS ions) assured the identification of the VIP compounds responsible for the differentiation of the two groups with high confidence. The suggested EC (elemental composition) for molecular ions and MS/MS fragments as well as the respective RDBeq (ring double bond equivalents) further assisted the safe identification process.

Generally, propolis samples are characterized by the presence of specific flavonoids [22,23,24,25,26,27,28]. Galangin, luteolin, chrysin, pinocembrin, pinobanksin, and two pinobanksin O-acetates are seven out of the ten most important VIPs that separate Greek samples from Chinese. The Greek propolis contains several of these flavonoids; however, the Chinese samples overexpress those particular flavonoids. All of these compounds were purchased as authentic samples and recorded in the BPI in-house library. Thus, those constituents were unambiguously identified in the unknown samples through comparison of the exact mass, Rt, RDBeq, and fragmentation mechanisms to the authentic standard (Table S1). All flavonoids were identified in the negative ESI mode.

Galangin’s concentration was remarkably lower in the Greek samples; thus, galangin is an important compound that differentiates the two groups. Galangin was the only flavonol overexpressed in Chinese samples. On the other hand, many flavones seem to characterize the Chinese samples. Apart from chrysin and luteolin, acetacin and another dihydroxyflavone were among the VIPs. Acacetin was also compared to the authentic standard for identification. The unknown dihydroxyflavone is eluted at 15.46 min and its MF (molecular formula) is C_15_H_10_O_4_. The fragmentation mechanism ends up to a characteristic MS/MS fragment at m/z 151, which corresponds to the detachment of the flavone B ring. The rest of the compounds were identified by comparison to the authentic standards. Some flavonols, such as quercetin, rhamnetin, isorhamnetin, apigenin, and two quercetin dimethylated analogs are overexpressed in the Greek samples. The first three flavonols were also compared with the authentic standards, which allowed for their safe identification. As for the quercetin analogs, their spectrometric features were analyzed. More specifically, for both compounds, after the detachment of the two methyl groups, the ion 301 (C_15_H_9_O_7_) is generated, which correspond to a quercetin aglycon.

Pinobanksin and several of its derivatives have been found to be major VIP constituents in the Chinese propolis samples. Pinobanksin is a flavanolol, biosynthesized by pinocembrin. Similar to pinocembrin, it is very common in bee products and propolis, especially of the poplar type. Interestingly, among the VIP compounds that differentiate the two groups, the Chinese samples contain, apart from pinobanksin and pinocembrin, two pinobanksin acetates and a pinobanksin isomer. All compounds were matched with the in-house library compounds. For the identification of their isomers, the fragmentation patterns were compared to those of the standards, where their structural similarity was confirmed. Remarkably, the Greek propolis samples overexpress—in comparison to the Chinese samples—two prenylated derivatives of pinobanksin and two prenylated derivatives of pinocembrin. The structures are confirmed by the study of the MS/MS, where the detachment of the prenyl group or O-prenyl is always a major fragment. Mass spectra were also compared with data from the literature. Prenylation of these compounds seems to be characteristic of the Greek samples. Additionally, the characteristic flavanone pinostrobin is expressed relatively more in the Greek samples (identified by comparison to the in-house library).

Hydroxylinolenic acid together with several other minor fatty acids, like stearic, arachidic, and eicosanoic acids, are found to be significant components in the Chinese samples but not in the Greek ones. The identification of these compounds was based on the MF given by the HR mass spectrometer as well as on the characteristic MS/MS fragmentation pattern of the fatty acids with the continuous detachments of CH_2_ groups. This type of compounds was among the top 10 VIPs that separate the two sample categories.

Furthermore, coumaroyl derivatives are common in propolis samples harvested from the Mediterranean region [22]. In this study, a coumaroyl derivative was detected as a VIP only in the propolis of Greek origin. The compound is eluted at 16.29 min and its MF is C_15_H_18_O_4_. The major MS/MS fragment is at m/z 177, its MF is C_10_H_9_O_3_, and it corresponds to a methoxycoumarin structure. Thus, the compound can be annotated as a derivative of methoxycoumarin. The Greek samples also overexpress two caffeoyl and phenyl compounds. The caffeic acid phenylethyl ester has been also confirmed with comparison to the authentic standard, while similar compounds have been described in the past [22] for propolis samples. In addition, a phenolic glucoside has been tentatively identified sharing same fragmentation patterns.

Another significant characteristic of the Greek propolis is the presence of terpenoids. Terpenes are a category of secondary metabolites that characterize propolis samples of the Mediterranean origin [22,23,24,25,26,29]. Diterpenes, triterpenes, and sesquiterpenes are predominant among the compounds that differentiate the Greek species. All terpenoids are detected in the positive ESI mode. Their identification was based on their exact molecular ions, their fragmentation mechanism, and all that in correlation to the literature. The sesquiterpenes have a characteristic C_15_H_24_ skeleton. Two sesquiterpenes were identified among the VIPs that characterize the Greek propolis. The first one was eluted at Rt of 16.78 min and corresponds to the MF C_15_H_22_O_2_. Its MS/MS fragmentation pattern confirms that the sesquiterpene is carboxylated, as the characteristic detachment of the carboxyl group leads to the fragment C_14_H_20_. After that, the skeleton is gradually fragmented, leading to the fragments 107 (C_8_H_11_), 95 (C_7_H_11_), and 81 (C_6_H_9_), common for more of the terpenoids. The compound could correspond to artemisinic acid, which is a molecule detected in samples of Mediterranean propolis in the past [22]. The second sesquiterpene is eluted at 19.55 min and its MF is C_16_H_23_O_2_, indicating the presence of an extra carbon in the skeleton. It also leads to a detachment of the carboxylic group initially, which also follows a gradual detachment of the skeleton.

In addition, diterpenes were one important category for the differentiation of the two groups, as two of them are among the top ten most significant VIPs. More specifically, pimaric and isopimaric acids, two isomer diterpenes characteristic of the *Pinus* species, seem to be major constituents of the Greek propolis samples and have been found before in propolis of the Mediterranean area [23,24]. Those two diterpenic acids have the same MF, C_20_H_31_O_2_, and give the characteristic detachment of the carboxyl group, followed by the gradual degradation of the diterpenic skeleton. Three more diterpenes were identified among VIPs, bearing similar C_20_ skeletons. The first one, eluted at Rt 19.22 min, has MF C_20_H_34_O_2_ and is tentatively identified as agathadiol based on data from the literature [23]. The other two diterpenes have a very close Rt (2.13 and 21.14 min) and they differentiate with one extra OH group. More specifically, the MF of the first one is C_20_H_32_O_2_, while the MF of the second one is C_20_H_34_O_3_. Both compounds share the similar fragmentation mechanism giving fragments at m/z 175 (C_13_H_19_), 149 (C_11_H_17_), 119 (C_9_H_11_), 107 (C_8_H_11_), 95 (C_7_H_11_), and 81 (C_6_H_9_). The latter is tentatively identified as imbricatoloic acid, a diterpenic acid found in propolis samples before [24,26]. Due to the similar Rt and fragmentation pattern, it is safe to suggest that the first diterpene is a hydroxylated isomer of imbricatoloic acid.

Among triterpenoids, two compounds that belong to this category were found among the comparison VIPs. One of them, with Rt of 31.94, was tentatively identified as an isomer of ursolic acid, which exists in the in-house library (Table S1). The MF and fragmentation mechanism are the same as of ursolic acid; however, there is a slight alteration in the Rt; thus, the unknown compound is mentioned as an ursolic acid isomer. The second triterpene detected is a major constituent of most Greek samples and one of the top ten VIPs. Its MF is C_30_H_47_O_2_, which is typical of a triterpene; however, the fragmentation mechanism does not exactly follow the ursolic acid (or any other triterpenic acid) (Table S1) pattern. Thus, it is rather identified as triterpenic alcohol rather than acid. One of the triterpenic alcohols found in propolis samples is ganoderol [29], which follows this kind of fragmentation as the unknown compound.

Finally, abscisic acid, a characteristic plant hormone found in stressed plants, is detected at Rt of 16.38 and characterizes the Greek group. This can be due to the climate conditions, as drought and other stresses trigger formation of this compound, especially in the Mediterranean region [22].

**Table 1:** Chromatographic and spectrometric features of the compounds responsible for the differentiation (VIPs) of Greek and Chinese propolis samples in the positive and negative ion modes.

Rt (min)	Ion Mode	m/z	Molecular Formula	RDBeq	MS/MS Fragments	Identification	Reference
***Overexpressed in Greek Propolis Samples***							
11.97	−	301.0425	C_15_H_9_O_7_	10.5	273 (C_14_H_9_O_6_), 229 (C_13_H_9_O_4_), 179 (C_8_H_3_O_5_), 151 (C_7_H_3_O_4_), 121 (C_7_H_5_O_2_), 107 (C_6_H_3_O_2_)	Quercetin	IHL *
12.32	−	329.0810	C_17_H_13_O_7_	10.5	315 (C_16_H_11_O_7_), 301 (C_15_H_9_O_7_), 269 (C_16_H_13_O_4_), 241 (C_15_H_13_O_3_), 167 (C_8_H_7_O_4_), 131 (C_9_H_7_O)	Quercetin dimethyl ether	
13.64	−	315.0513	C_16_H_11_O_7_	11.5	300 (C_15_H_8_O_7_), 165 (C_8_H_5_O_4_), 121 (C_7_H_5_O_2_)	Isorhamnetin	IHL
13.79	−	301.1082	C_16_H_13_O_6_	9.5	286 (C_13_H_10_O_6_), 258 (C_15_H_14_O_4_), 164 (C_8_H_4_O_4_), 151 (C_7_H_3_O_4_)	Hesperetin	IHL
14.62	−	315.0512	C_16_H_11_O_7_	11.5	300 (C_15_H_8_O_7_), 271 (C_15_H_11_O_5_), 253 (C_15_H_9_O_4_), 165 (C_8_H_5_O_4_)	Rhamnetin	IHL
14.97	−	269.0812	C_14_H_15_O_4_	10.5	225 (C_14_H_9_O_3_), 151 (C_7_H_3_O_4_), 117 (C_6_H_5_O)	Apigenin	IHL
15.26	+	331.0809	C_17_H_15_O_7_	10.5	316 (C_16_H_12_O_7_), 301 (C15H9O7), 273 (C_14_H_9_O_6_), 151 (C_8_H_7_O_3_), 137 (C_7_H_5_O_3_)	Quercetin dimethyl ether	[16]
15.27	−	247.0975	C_14_H_15_O_4_	7.5	179 (C_9_H_7_O_4_), 161 (C_9_H_5_O_3_), 139 (C_7_H_7_O_3_), 137 (C_7_H_5_O_3_)	Phenyl caffeate	[22]
15.53	−	283.0967	C_17_H_15_O_4_	10.5	268 (C_15_H_8_O_5_), 239 (C_14_H_7_O_4_), 179 (C_9_H_7_O_4_), 161 (C_9_H_5_O_3_), 135 (C_8_H_7_O_2_)	Phenethyl caffeate	IHL
16.27	+	263.1276	C_15_H_19_O_4_	6.5	177 (C_10_H_9_O_3_), 145 (C_9_H_5_O_2_), 117 (C_8_H_5_O)	Methoxycoumarin derivative	
16.38	−	265.1445	C_15_H_21_O_4_	5.5	221 (C_14_H_21_O_2_), 99 (C_5_H_7_O_2_)	Abscisic acid	[25]
16.44	+	195.0651	C_10_H_11_O_4_	5.5	177 (C_10_H_9_O_3_), 163 (C_9_H_7_O_3_), 149 (C_9_H_9_O_2_), 145 (C_9_H_5_O_2_)	Cinnamoyl derivative	-
16.78	+	234.1605	C_15_H_23_O_2_	4.5	189 (C_14_H_21_), 161 (C_12_H_17_), 147 (C_11_H_15_), 133 (C_10_H_13_), 107 (C_8_H_11_), 95 (C_7_H_11_), 81 (C_6_H_9_)	Sesquiterpene acid, e.g., artemisinic acid	[22]
17.71	+	271.0961	C_16_H_15_O_4_	9.5	167 (C_8_H_7_O_4_), 131 (C_9_H_7_O), 103 (C_8_H_7_)	Pinostrobin	IHL
19.22	+	307.2630	C_20_H_35_O_2_	3.5	149 (C_11_H_17_), 109 (C_8_H_13_), 95 (C_7_H_11_), 81 (C_6_H_9_)	Diterpene, e.g., agathadiol	[23]
19.58	+	303.2316	C_20_H_31_O_2_	5.5	257 (C_19_H_29_), 201 (C_15_H_21_), 147 (C_11_H_15_), 119 (C_9_H_11_), 105 (C_8_H_9_), 81 (C_6_H_9_), 67 (C_5_H_7_)	Pimaric/isopimaric acid **	[24]
19.78	+	303.2318					
19.17	+	357.1330	C_20_H_21_O_6_	11.5	273 (C_15_H_13_O_5_), 255 (C_15_H_11_O_4_), 227 (C_14_H_11_O_3_), 199 (C_13_H_11_O_2_), 153 (C_7_H_5_O_4_) (pos); 253 (C_15_H_9_O_4_), 225 (C_14_H_9_O_3_), 151 (C_7_H_3_O_4_), (neg)	Hydroxyl pinobanksin-O-pentanoate or hydroxyl pinobanksin-O-methyl butyrate	[16]
19.50	±	357.1332; 355.1321					
19.55	+	247.1691	C_16_H_23_O_2_	5.5	229 (C_16_H_21_O), 201 (C_15_H_21_), 173 (C_13_H_17_), 159 (C_12_H_15_), 145 (C_11_H_13_)	Carboxyl sesquiterpenoid	
21.13	+	305.2474	C_20_H_33_O_2_	4.5	287 (C_20_H_31_O), 175 (C_13_H_19_), 161 (C_12_H_17_), 149 (C_11_H_17_), 119 (C_9_H_11_), 107 (C_8_H_11_), 95 (C_7_H_11_), 81 (C_6_H_9_)	Diterpene	[23]
21.14	+	325.2528	C_20_H_35_O_3_	4.5	175 (C_13_H_19_), 149 (C_11_H_17_), 119 (C_9_H_11_), 107 (C_8_H_11_), 95 (C_7_H_11_), 81 (C_6_H_9_)	Diterpenic acid, e.g., imbricatoloic acid	[24,26]
22.06	+	451.2090	C_22_H_27_O_10_	10.5	105 (C_8_H_9_), 91 (C_7_H_7_), 81 (C_6_H_9_)	Phenolic glycoside, e.g., torachrysone-O-galloyl hexose	[27]
23.18	+	325.1365	C_20_H_21_O_4_	10.5	257 (C_15_H_15_O_4_), 153 (C_7_H_5_O_4_), 131 (C_9_H_7_O), 103 (C_8_H_7_)	Pinocembrin, O-prenylated	[28]
25.67	+	339.1462	C_21_H_23_O_4_	10.5	271 (C_16_H_15_O_4_), 167 (C_8_H_7_O_4_), 131 (C_9_H_7_O), 103 (C_8_H_7_)	Pinocembrin methyl ether, O-prenylated	[28]
28.70	+	439.3532	C_30_H_47_O_2_	7.5	203 (C_15_H_23_), 189 (C_14_H_21_), 161 (C_12_H_1_7), 133 (C_10_H_13_), 119 (C_9_H_11_), 95 (C_7_H_11_), 81 (C_6_H_9_)	Triterpene, e.g., ganoderol A	[29]
31.94	+	455.3402	C_30_H_47_O_3_	7.5	409 (C_29_H_45_O), 203 (C_15_H_23_), 189 (C_14_H_21_), 175 (C_13_H_19_), 147 (C_11_H_15_), 121 (C_9_H_13_)	Triterpenic acid (ursolic acid isomer)	[22], IHL
***Overexpressed in Chinese Propolis Samples***							
8.45	−	163.0512	C_9_H_5_O_3_	6.5	119 (C_8_H_7_O), 117 (C_8_H_5_O)	Coumaric acid	IHL
12.31	+	287.0834	C_15_H_11_O_6_	10.5	269 (C_15_H_9_O_5_), 241 (C_14_H_9_O_4_), 171 (C_7_H_7_O_5_), 153 (C_7_H_5_O_4_), 137 (C_7_H_5_O_2_)	Luteolin	IHL
12.71	±	273.0613; 271.0584	C_15_H_13_O_5_; C_15_H_11_O_5_	10.5	255 (C_15_H_11_O_4_), 227 (C_14_H_11_O_3_), 199 (C_13_H_11_O_2_), 153 (C_7_H_5_O_4_) (pos); 253 (C_15_H_9_O_4_), 225 (C_14_H_9_O_3_), 215 (C_13_H_11_O_3_), 197 (C_13_H_9_O_2_), 161 (C_10_H_9_O_2_), 151 (C_7_H_3_O_4_), 125 (C_6_H_5_O_3_), 107 (C_6_H_3_O_2_), 83 (C_4_H_3_O_2_) (neg)	Pinobanksin	IHL
14.49	±	315.0861; 313.0791	C_17_H_15_O_6_; C_17_H_13_O_6_	11.5	300 (C_16_H_12_O_6_), 271 (C_15_H_11_O_5_), 255 (C_14_H_7_O_5_), 243 (C_13_H_7_O_5_) (neg)	Pinobanksin O-acetate	IHL
14.89	−	255.0693	C_15_H_13_O_4_	11.5	227 (C_14_H_11_O_3_), 213 (C_13_H_9_O_3_), 171 (C_11_H_7_O_2_), 151 (C_7_H_3_O_4_), 107 (C_6_H_3_O_2_), 83 (C_4_H_3_O_2_)	Pinocembrin	IHL
15.46	−	253.0586	C_15_H_9_O_4_	11.5	151 (C_7_H_3_O_4_), 107 (C_6_H_3_O_2_), 83 (C_4_H_3_O_2_)	Dihydroxyflavone	
15.15	±	315.0837; 313.0789	C_17_H_15_O_6_; C_17_H_13_O_6_	11.5	255 (C_15_H_11_O_4_), 227 (C_14_H_11_O_3_), 199 (C_13_H_11_O_2_), 153 (C_7_H_5_O_4_) (pos); 300 (C_16_H_12_O_6_), 271 (C_15_H_11_O_5_), 253 (C_15_H_9_O_4_), 225 (C_14_H_9_O_3_), 197 (C_13_H_9_O_2_), 151 (C_7_H_3_O_4_) (neg)	Pinobanksin 3-O-acetate	IHL
16.03	±	271.0573; 269.0493	C_15_H_11_O_5_; C_15_H_9_O_5_	11.5	215 (C_13_H_11_O_3_), 197 (C_13_H_9_O_2_), 165 (C_8_H_5_O_4_), 153 (C_7_H_5_O_4_), 131 (C_9_H_7_O, 105 (C_7_H_5_O) (pos); 241 (C_14_H_9_O_4_), 227 (C_13_H_7_O_4_), 213 (C_13_H_9_O_3_), 197 (C_13_H_9_O_2_), 169 (C_12_H_9_O), 143 (C_10_H_7_O) (neg)	Galangin	IHL
15.63	+	255.0632	C_15_H_11_O_4_	10.5	225 (C_10_H_9_O_6_), 153 (C_7_H_5_O_4_)	Chrysin	IHL
15.15	+	273.0613	C_15_H_13_O_5_	10.5	227 (C_14_H_11_O_3_), 199 (C_13_H_11_O_2_), 153 (C_7_H_5_O_4_)	Flavanol, pinobanksin isomer, e.g., garbanzol	IHL
16.36	+	285.0803	C_16_H_13_O_5_	10.5	242 (C_14_H_10_O_4_), 153 (C_7_H_5_O_4_), 105 (C_8_H_9_)	Acacetin	IHL
22.39	+	295.2212	C_18_H_31_O_3_	3.5	165 (C_11_H_17_O), 151 (C_10_H_15_O), 133 (C_10_H_13_), 107 (C_8_H_11_), 95 (C_6_H_7_O), 81 (C_5_H_5_O), 67 (C_5_H_7_)	Hydroxylinolenic acid	
25.31	+	-	-	-	-	Fatty acids (stearic, arachidic, eicosanoic acid derivatives)	

* In-house library (IHL). List of standard compounds can be found in the Supplementary materials, Table S1. ** Compounds highlighted in bold are among the top ten VIP metabolites. Box plots and structures of compounds can be found in the Supplementary materials, Figures S6 and S8.

## 3. Conclusions

In continuation of our research on propolis [9,10] and following early findings on geographical differentiation, untargeted LC-MS metabolomics analysis was employed for chemical composition comparison of Greek and Chinese propolis extracts. Applying PCA, two groups were straightforwardly separated, Greek and Chinese samples, both in the positive and the negative ion modes. The clear separation of clusters in the unsupervised PCA model suggest phytochemical differences between the samples harvested from Greece and China. In addition, supervised models PLS-DA and OPLS-DA were also applied, showing statistically significant separation between the two groups.

Comparing the chemical profiling of all samples, compounds responsible for group clustering were further investigated. In more detail, after construction of valid chemometric models, it was observed that the Chinese samples overexpress galangin, which could be marked as an important compound that differentiates the two groups. Moreover, several flavones, such as chrysin, acacetin, and luteolin, belong to the compounds that are overexpressed in the Chinese samples. Fatty acids, such as hydroxylinolenic, stearic, arachidic, and eicosanoic acid derivatives are found as significant components in the Chinese samples. All of these components are characteristic of the poplar type propolis, which is common in the temperate regions of Europe, North America, and China [16]. The obtained results match those of the study of Wang et al. [11], where it was observed that the main plant origin of Chinese propolis is *P. canadensis*. It is important to note that among the VIP compounds that differentiate the two groups, the Chinese samples contain pinobanksin, pinocembrin, two pinobanksin acetates, and a pinobanksin isomer, whereas Greek propolis samples overexpress two prenylated derivatives of pinobanksin and two prenylated derivatives of pinocembrin. Prenylation of these compounds seems to be characteristic of Greek propolis samples.

Moreover, flavonoids such as quercetin, dimethylated quercetin, and pinobanksin-O-pentanoate and some aromatic acids, such as ferulic acids and caffeic acid esters, which are dominant compounds in Greek propolis, are also in accordance with the literature on the poplar type propolis [30]. Greek propolis also overexpresses terpenoids; diterpenes, triterpenes, and sesquiterpenes are predominant, in particular, diterpenes such as pimaric/isopimaric acid, agathadiol, imbricatoloic acid, etc., which are characteristic of the Mediterranean propolis type [31]. Their source could be some conifer species of the Cupressaceae family, in which the flora of the region is known to be rich [8]. Besides Mediterranean propolis, Brazilian propolis from *Araucaria* species is also rich in labdane diterpenic acids [32]. What is also interesting about Greek samples is the presence of abscisic acid, which is a plant hormone. Large amounts of abscisic acid have been found in the Po Valley samples of Northern Italy, possibly due to great plant exposure to stress (high temperature, dryness, etc.) [22]. Finally, it is important to note that coumaroyl derivatives are found both in Greek and Chinese extracts. Their botanical origin is yet unidentified. They are common in propolis samples harvested from the Mediterranean region [22], but they have also been reported in *Populus* type propolis coming from Belarus, Poland, Russia, Turkey, and Bulgaria [30].

In conclusion, untargeted LC-MS metabolomics analysis of Greek and Chinese propolis samples from different regions revealed significant differences between them. The results demonstrated high potential for construction of geographical mapping, which would allow for easier standardization of propolis, something necessary to guarantee quality, efficacy, and safety of propolis-based pharmaceutical products.

## 4. Materials and Methods

### 4.1. Chemicals, Reagents, Analytical Standards, and Instrumentation

Solvents for propolis extraction (n-heptane and methanol) were of analytical grade, purchased from Merck (Merck, Darmstadt, Germany). LC-MS-grade methanol (MeOH) and formic acid (FA) were purchased from Fisher Scientific (Fisher Optima, Waltham, MA, USA) and LC-MS water was produced using an SG Millipore apparatus.

Caffeic acid, caffeic acid phenethyl ester (CAPE), chrysin, luteolin, daidzein, suberic acid, apigenin were obtained from Alfa Aesar, Pittsburg, PA, USA. Pinocembrin, isorhamnetin, isosakuranetin, vitexin, orientin, rosmarinic acid, vanillin, ursolic acid, chrysoeriol, betulinic acid, eriodictyol, sakuranetin, naringenin, genistein, diosmetin, resveratrol, galangin, pinocembrin 7-methyl ether were purchased from Extrasynthese, Genay Cedex, France. Isoferulic acid, kaempferol, quercetin, corosolic acid, acacetin, diosmin, protocatechuic acid ethyl ester, hesperetin, phloridzin, chlorogenic acid, p-coumaric acid, maslinic acid were bought from Sigma Aldrich, St. Louis, MO, USA. Rhamnetin, protocatechuic acid, ferulic acid, kaempferide, adipic acid, pinostrobin, pinobanksin were purchased from Fluka, St. Louis, MO, USA; naringin and hesperidin—from Acros Organics, Morris Plains, NJ, USA; and pinobanksin-3-O-acetate—from Interchim Inc., Los Angeles, CA, USA.

The ultrahigh-performance liquid chromatography was performed employing a Dionex Ultimate 3000 UHPLC system (Thermo Scientific, Waltham, MA, USA) equipped with a binary pump, an autosampler, an online vacuum degasser, and a temperature-controlled column compartment. A Hypersil Gold UPLC C18 (2.1 × 150 mm, 1.9 μm) reversed-phase column (Thermo Scientific, Germany) was used for the analysis. The high-resolution mass spectrometry was performed on a Orbitrap Q-Exactive mass spectrometer (Thermo Scientific, Germany).

### 4.2. Sample Collection and Extract Preparation

Twenty-two propolis samples were collected from eleven different regions of Greece and twenty-three samples were collected from sixteen different regions of China as indicated in Table 2. All samples were stored at −20 °C until analysis. Voucher specimens are deposited in the Department of Pharmacognosy and Natural Products Chemistry, Faculty of Pharmacy, National and Kapodistrian University of Athens, University of Athens, Greece. All samples were grounded prior to extraction with the same extraction protocol. Approximately 4 g of each sample were initially defatted with n-heptane (2 × 20 mL) and subsequently the remaining material was extracted in an ultrasonic bath with methanol (2 × 20 mL). The methanolic extracts were merged, concentrated to dryness by a rotary evaporator (Büchi, Switzerland) under vacuum. Six different parts of each propolis sample were treated according to the above procedure resulting in the preparation of six extracts. Three extracts were selected randomly and forwarded for LC-MS analysis. Dry extracts were stored at −20 °C until analysis.

### 4.3. UHPLC-HRMS Conditions

Samples were injected at concentration of 100 ppm diluted in MeOH/H2O (50:50). Equal aliquots (5 μL) of the studied samples were thoroughly mixed and the pooled samples derived were used as QC samples. The mobile phase consisted of solvents A (aqueous 0.1% (*v*/*v*) formic acid) and B (methanol). Different gradient elutions were performed for positive and negative ion mode detection and after optimization of the chromatography, the gradients applied were as follows: T = 0 min, 20% B; T = 2 min, 20% B; T = 12 min, 70% B; T = 32 min, 95% B; T = 37 min, 95% B; T = 37.1 min, 20% B; T = 40 min, 20% B. The flow rate was 0.220 mL/min and the injection volume was 5 μL. The column temperature was kept at 40 °C while the sample tray temperature was set at 10 °C. The ionization was performed using heated ESI (HESI), both positive and negative modes. The conditions for the HRMS for both the negative and positive ionization modes were set as follows: capillary temperature, 350 °C; spray voltage, 2.7 kV; S-lens Rf level, 50 V; sheath gas flow, 40 arb. units; auxiliary gas flow, 5 arb. units; auxiliary gas heater temperature, 50 °C. Analysis was performed using the Fourier-transform mass spectrometry mode of the linear trap quadrupole (LTQ) Orbitrap (FTMS) in the full scan ion mode, applying a resolution of 70,000, while acquisition of the mass spectra was performed in every case using the centroid mode. The data-dependent acquisition capability was also used at the resolution of 35,000, allowing for MS/MS fragmentation of the three most intense ions of every peak exceeding the predefined threshold applying a 10 s dynamic exclusion. Stepped normalized collision energy was set at 40, 60, and 100. Data acquisition and analysis were completed employing Xcalibur 2.1.

### 4.4. Orbitrap-Based Metabolomics and Chemometrics Data Analysis

Data were imported to Compound Discoverer 2.1 (Thermo Scientific) prior to statistical analysis. The workflow was an untargeted metabolomics approach for detecting, among other things, unknown compounds, mapped pathways and searching in online databases. The appropriate set-up for peak detection, deconvolution, deisotoping, alignment, and gap filling procedures was applied. For the gap filling, the signal-to-noise threshold was set at 1.5. The normalization was QC-based and the regression model used was linear. Blanks were excluded and the type was constant mean. The appropriate settings were also used for the composition prediction, setting the mass tolerance at 5 ppm. The peak annotation was performed in comparison to online spectral libraries. The mass tolerance was also set at 5 ppm. In addition, a mass list by injecting 45 authentic standards found in propolis was used for dereplication purposes (Table S1). Furthermore, for the structural identification of the constituents and the annotation of the peaks, m/z vault and m/z cloud applications were included in the structural elucidation process with a specialization in natural product databases and a Fourier transform (FT) fragment’s mass tolerance of 10 ppm. The generated peak list (accurate mass vs. intensity vs. predicted compositions) was exported as a .csv file to Microsoft Excel and manipulated appropriately using the CONCATENATE, ROUND, and TRANSPOSE commands.

The data obtained by MS were analyzed employing multivariate statistical analysis. The PCA, PLS-DA, and OPLS-DA models were constructed with the Compound Discoverer and SIMCA (Umetrics, Sweden) software. Principal component analysis (PCA) was used to examine the quality characteristics of the method by visual inspection of the QC clustering and partial least Squares discriminant analysis (PLS-DA) was used to determine any possible classification between the examined groups, as well as to explore the important features responsible for that clustering. In order to validate the statistical models, permutation testing was performed employing 100 random permutations for the PLS-DA and OPLS-DA analyses. The optimal number of principal components employed was determined by minimizing the difference between Q2 and R2 for all multivariate analysis (MVA) models used. The variable importance in projection (VIP) values were used to indicate the most influential parameters for the classification of the groups. The VIPs calculated for the first principal component (PC1) of the PLS-DA models were selected when values were > 1.5 for the positive mode and > 1.1 for the negative mode.

## Figures and Tables

**Table 2:** Coding of propolis samples collected from Greece and China. The year of harvest was 2013–2015 for all samples. Harvest period details were provided only for Greek samples.

Propolis Code	Origin	Season/year
	**Origin (Greece)**	
PR_1	Arta, Epirus	Fall 2013
PR_2	Arta (lowland), Epirus	Fall 2013
PR_3	Mt. Olympus, Macedonia	Fall 2013
PR_4	Drama, Macedonia	Fall 2013
PR_5	Arta, Epirus	Fall 2013
PR_6	Serres, Macedonia	Fall 2013
PR_7	Chania, Crete	Fall 2013
PR_8	Euboea Island	Fall 2013
PR_9	Mt. Olympus, Macedonia	Spring 2013
PR_10	Samos Island	Fall 2013
PR_11	Samos Island	Fall 2013
PR_12	Kopaida, Boeotia	Fall 2013
PR_13	Serres, Macedonia	Fall 2014
PR_14	Mt. Olympus, Macedonia	Summer 2014
PR_15	Mt. Olympus, Macedonia	Spring 2014
PR_16	Evros, Thrace	Summer 2014
PR_17	Naousa, Macedonia	Summer 2014
PR_18	Markopoulo, Attica	Summer 2014
PR_19	Markopoulo, Attica	Summer 2014
PR_20	Kopaida, Boeotia	Summer 2014
PR_21	Chora, Samothraki	Spring 2015
PR_22	Makrilies, Samothraki	Spring 2015
	**Origin (China)**	
PC_1	Yili, Xinjiang Autonomous Region	
PC_2	Hainan Region	
PC_3	Longchang, Sichuan Province	
PC_4	Xi’an, Shanxi Province	
PC_5	Fuzhou, Fujian Province	
PC_6	Lijiang, Yunnan Province	
PC_7	Zunyi, Guizhou Province	
PC_8	Raohe, Heilongjiang Province	
PC_9	Yongzhou, Hunan Province	
PC_10	Xining, Qinghai Province	
PC_11	Zaoyang, Hubei Province	
PC_12	Ulanhot, Neimenggu	
PC_13	Jiujiang, Jiangxi Province	
PC_14	Jianshi, Hubei Province	
PC_15	Changge, Henan Province	
PC_17	Jining, Shandong Province	
PC_18	Tianshui, Gansu Province	
PC_19	Maoming, Guangdong Province	
PC_23	not labeled	
PC_25	Changsha, Hunan Province	
PC_27	Xishuangbanna, Yunnan Province	
PC_28	Loudi, Hunan Province	
PC_29	Zhengzhou, Henan Province	

## Data Availability

Data is contained within the article or supplementary material. Any further information presented in this study is available on request from the corresponding author.

## Supplementary Materials

Figure S1: LC-MS chromatograms of Quality Control (QC) samples at negative (top) and positive (bottom) ion mode, Figure S2: For data acquired in positive ion mode. Top: PLS-DA model goodness of fit and prediction values. Bottom: PLS-DA model validation after 100 permutation tests for Greek and Chinese classified samples, Figure S3: For data acquired in positive ion mode. Top: OPLS-DA model goodness of fit and prediction values. Bottom: PLS-DA model validation after 100 permutation tests for Greek and Chinese classified samples; Figure S4: For data acquired in negative ion mode. Top: PLS-DA model goodness of fit and prediction values. Bottom: PLS-DA model validation after 100 permutation tests for Greek and Chinese classified samples; Figure S5: For data acquired in negative ion mode. Top: OPLS-DA model goodness of fit and prediction values. Bottom: OPLS-DA model validation after 100 permutation tests for Greek and Chinese classified samples, Figure S6: Box plots and variable importance in projection (VIPs) values extracted from the PLS-DA analysis revealed the most important features in negative (left) and positive (right) ESI, Figure S7: Base peak LC-MS chromatogram of the standard mixture at negative and positive mode (upper and lower chromatogram respectively); Figure S8: Structures of key compounds responsible for the differentiation (VIPs) of Greek and Chinese propolis samples in positive and negative ion mode according to Table 1, Table S1: List of the standard compounds’ mixture injected for dereplication purposes in negative ion mode (exact mass and molecular formula of m/z ion). Compounds are shorted according to their elution order with noted their spectrometric and chromatographic features.
