# Supplementary material for: Untargeted Ultrahigh-Performance Liquid Chromatography-Hybrid Quadrupole-Orbitrap Mass Spectrometry (UHPLC-HRMS) Metabolomics Reveals Propolis Markers of Greek and Chinese Origin
Source: Molecules. 2021 Jan 16;26(2):456. doi: 10.3390/molecules26020456 (PMC7830967; doi:10.3390/molecules26020456)
Supplement: Supplementary file 1 [file molecules-26-00456-s001.pdf]

molecules

MDPI

Supplementary material

Untargeted Ultrahigh-Performance Liquid Chromatography-Hybrid Quadrupole-Orbitrap Mass Spectrometry (UHPLC-HRMS) Metabolomics Reveals Propolis Markers of Greek and Chinese Origin

Maria-Ioanna Stavropoulou [1], Aikaterini Termentzi [2], Konstantinos M. Kasiotis [2], Antigoni Cheilari [1], Konstantina Stathopoulou [1], Kyriaki Machera [2] and Nektarios Aligiannis [1,]*

[1] Department of Pharmacognosy and Natural Products Chemistry, Faculty of Pharmacy, National and Kapodistrian University of Athens, Panepistimiopolis Zografou, Athens 11527, Greece; mstavropoul@yahoo.gr (M.-I.S.); cheilarianti@pharm.uoa.gr (A.C.); kstatho@pharm.uoa.gr (K.S.); aligiannis@pharm.uoa.gr (N.A)

[2] Laboratory of Pesticides' Toxicology, Department of Pesticides Control and Phytopharmacy, Benaki Phytopathological Institute, 8 St. Delta Street, Kifissia, Athens 14561, Greece; a.termentzi@gmail.com (A.T.); K.Kasiotis@bpi.gr (K.M.K.); k.machera@bpi.gr (K.M.)

* Correspondence: aligiannis@pharm.uoa.gr, Tel: +302107274524

Received: 30 November 2020; Accepted: 11 January 2021; Published: 16 January 2021

Abstract: Chemical composition of propolis depends on the plant source and thus on the geographic and climatic characteristics of the site of collection. The aim of this study was to investigate the chemical profile of Greek and Chinese propolis extracts from different regions and suggest similarities and differences between them. Untargeted ultrahigh-performance liquid chromatography coupled to hybrid quadrupole-Orbitrap mass spectrometry (UHPLC-HRMS) method was developed and 22 and 23 propolis samples from Greece and China, respectively, were analyzed. The experimental data led to the observation that there is considerable variability in terms of quality of the distinctive propolis samples. Partial least squares - discriminant analysis (PLS-DA) and orthogonal partial least squares - discriminant analysis (OPLS-DA) models were constructed and allowed the identification of significant features for sample discrimination, adding relevant information for the identification of class-determining metabolites. Chinese samples overexpressed compounds that are characteristic of the poplar type propolis, whereas Greek samples overexpress the latter and the diterpenes characteristic of the Mediterranean propolis type.

Keywords: Mediterranean propolis; poplar propolis; Greek propolis; Chinese propolis; UHPLC-HRMS; chemometrics

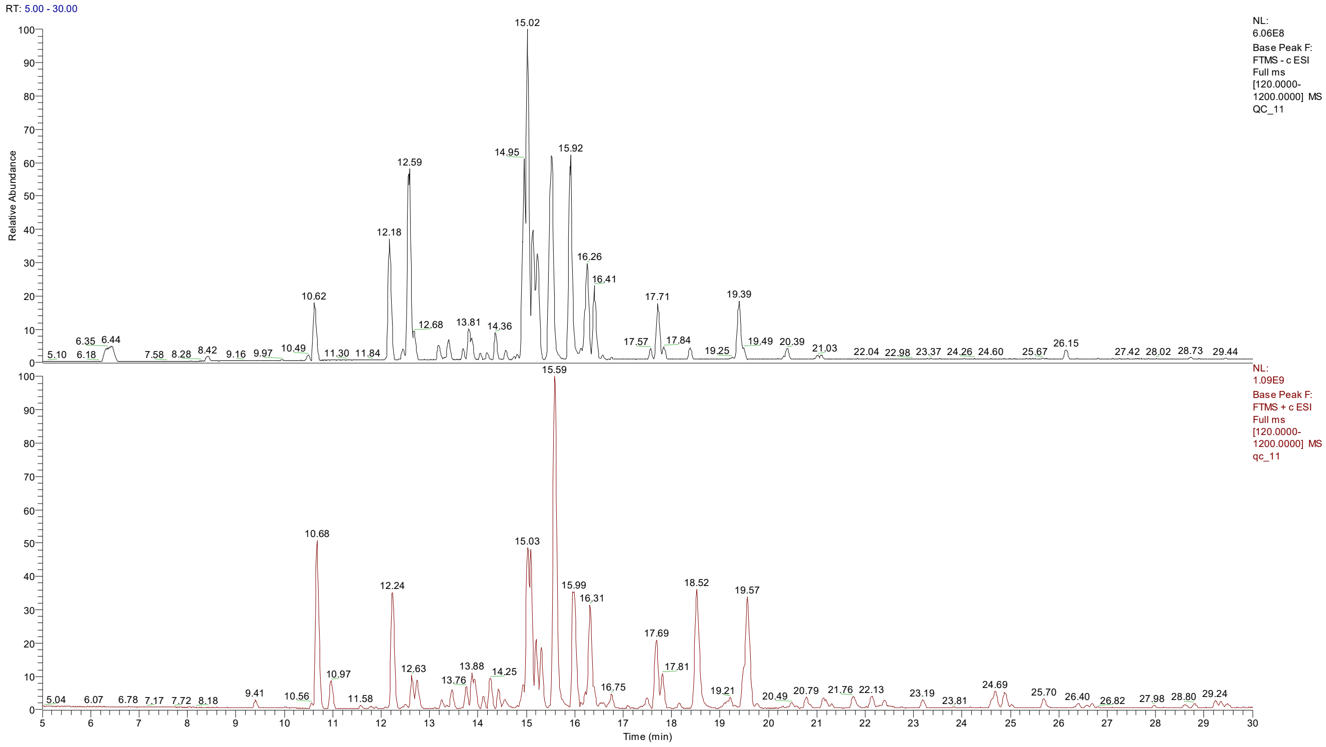

Figure S1: LC-MS chromatograms of Quality Control (QC) samples at negative (top) and positive (bottom) ion mode.

Component	R2X	R2X(cum)	Eigenvalue	R2Y	R2Y(cum)	Q2	Limit	Q2(cum)	Significance	Iterations
0	Cent.									
1	0.218	0.218	22	0.811	0.811	0.793	0	0.793	R1	2
2	0.136	0.354	13.7	0.133	0.944	0.677	0	0.933	R1	2
3	0.0414	0.396	4.18	0.0315	0.976	0.361	0	0.957	R1	2
4	0.0584	0.454	5.9	0.0104	0.986	0.28	0	0.969	R1	2

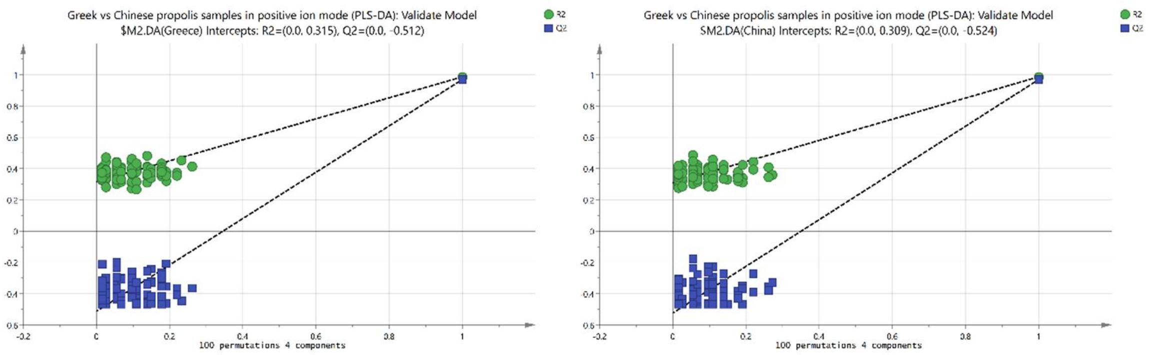

Figure S2: For data acquired in positive ion mode. ***Top***: PLS-DA model goodness of fit and prediction values. ***Bottom***: PLS-DA model validation after 100 permutation tests for Greek and Chinese classified samples.

Component	R2X	R2X(cum)	Eigenvalue	R2	R2(cum)	Q2	Limit	Q2(cum)	R2Y	R2Y(cum)	EigenvalueY	Significance
Model		0.454			0.986			0.971		1		
Predictive		0.162			0.986			0.971		1		
P1	0.162	0.162	16.4	0.986	0.986	0.971	0.01	0.971	1	1	2	R1
Orthogonal in X(OP...		0.292			0							
O1	0.184	0.184	18.6	0	0							R1
O2	0.0474	0.232	4.79	0	0							R1
O3	0.0604	0.292	6.1	0	0							R1

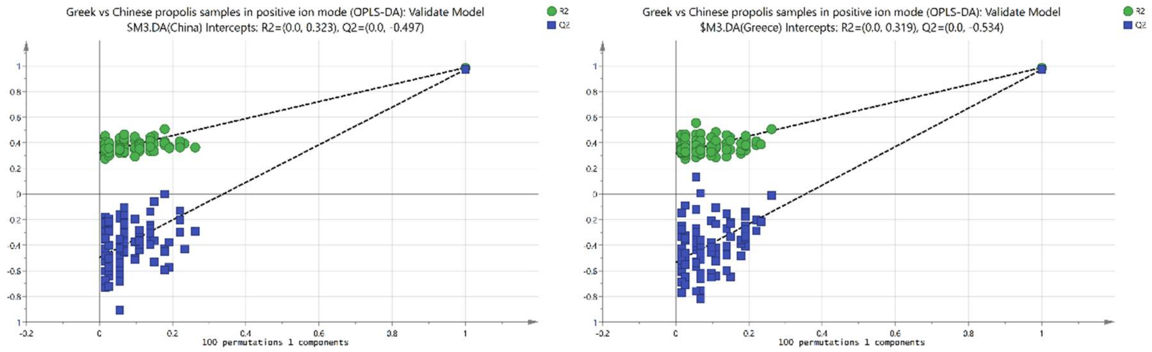

Figure S3: For data acquired in positive ion mode. ***Top***: OPLS-DA model goodness of fit and prediction values. ***Bottom***: PLS-DA model validation after 100 permutation tests for Greek and Chinese classified samples.

Component	R2X	R2X(cum)	Eigenvalue	R2Y	R2Y(cum)	Q2	Limit	Q2(cum)	Significance	Iterations
0	Cent.									
1	0.181	0.181	18.2	0.829	0.829	0.808	0	0.808	R1	2
2	0.147	0.328	14.9	0.0603	0.889	0.307	0	0.867	R1	2
3	0.0736	0.402	7.44	0.0498	0.939	0.308	0	0.908	R1	2
4	0.0751	0.477	7.58	0.0253	0.964	0.293	0	0.935	R1	2
5	0.0618	0.538	6.24	0.0112	0.975	0.0847	0	0.94	R1	2

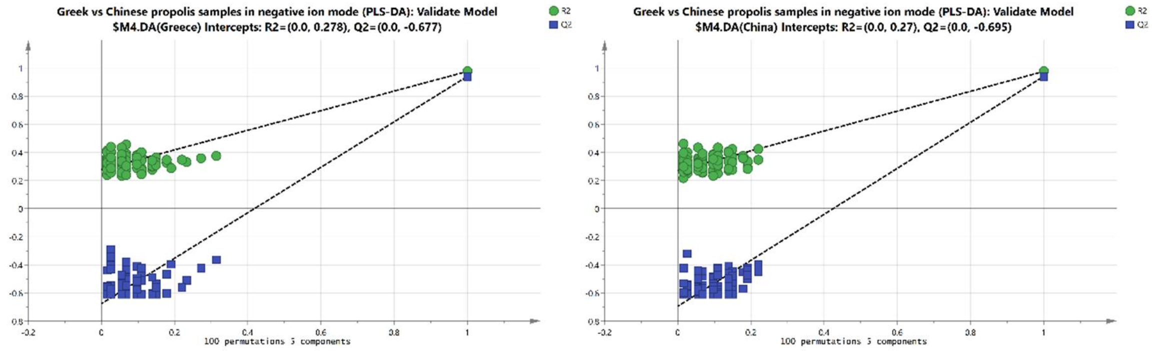

Figure S4: For data acquired in negative ion mode. ***Top***: PLS-DA model goodness of fit and prediction values. ***Bottom***: PLS-DA model validation after 100 permutation tests for Greek and Chinese classified samples.

Component	R2X	R2X(cum)	Eigenvalue	R2	R2(cum)	Q2	Limit	Q2(cum)	R2Y	R2Y(cum)	EigenvalueY	Significance
Model		0.477			0.964			0.934		1		
Predictive		0.149			0.964			0.934		1		
P1	0.149	0.149	15	0.964	0.964	0.934	0.01	0.934	1	1	2	R1
Orthogonal in X(OP...		0.328			0							
O1	0.165	0.165	16.6	0	0							R1
O2	0.0829	0.248	8.38	0	0							R1
O3	0.0803	0.328	8.11	0	0							R1

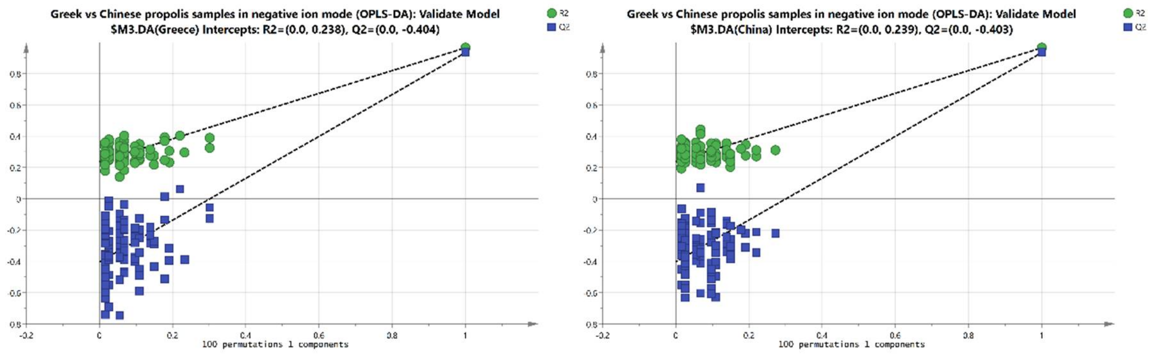

Figure S5: For data acquired in negative ion mode. ***Top***: OPLS-DA model goodness of fit and prediction values. ***Bottom***: OPLS-DA model validation after 100 permutation tests for Greek and Chinese classified samples.

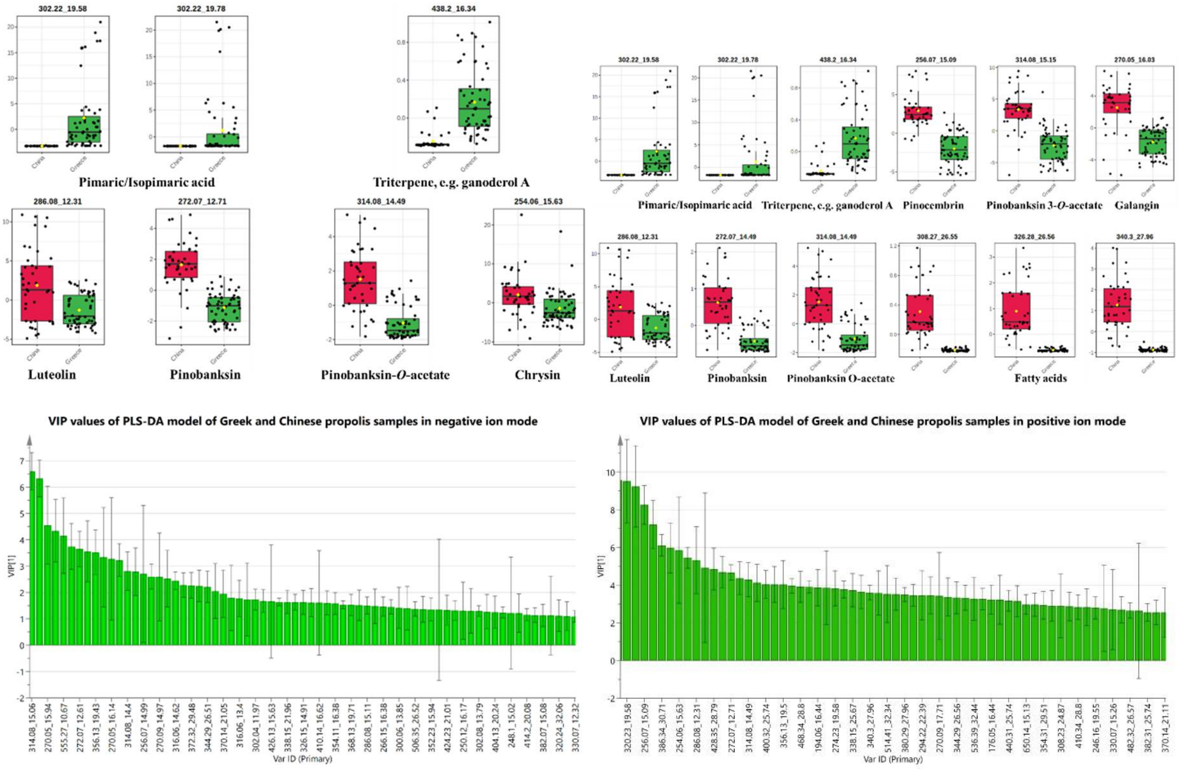

Figure S6: Box plots and variable importance in projection (VIPs) values extracted from the PLS-DA analysis revealed the most important features in negative (left) and positive (right) ESI.

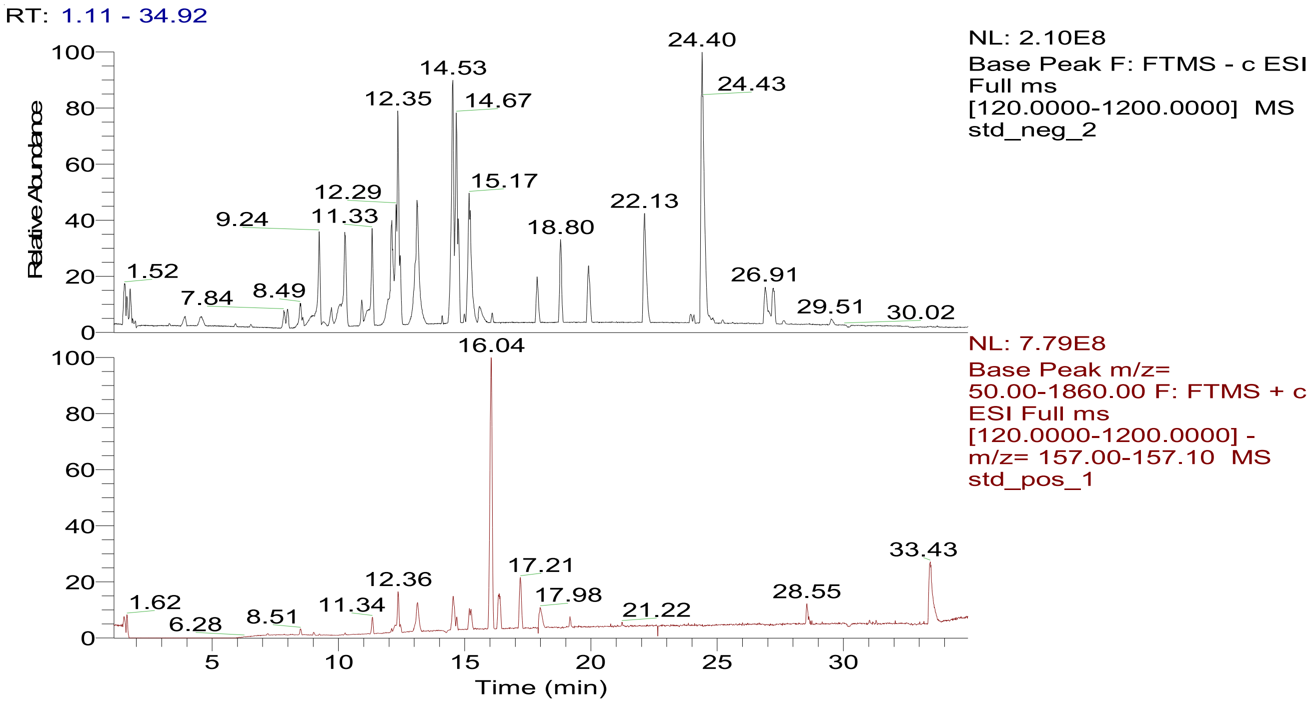

Figure S7: Base peak LC-MS chromatogram of the standard mixture at negative and positive mode (upper and lower chromatogram respectively).

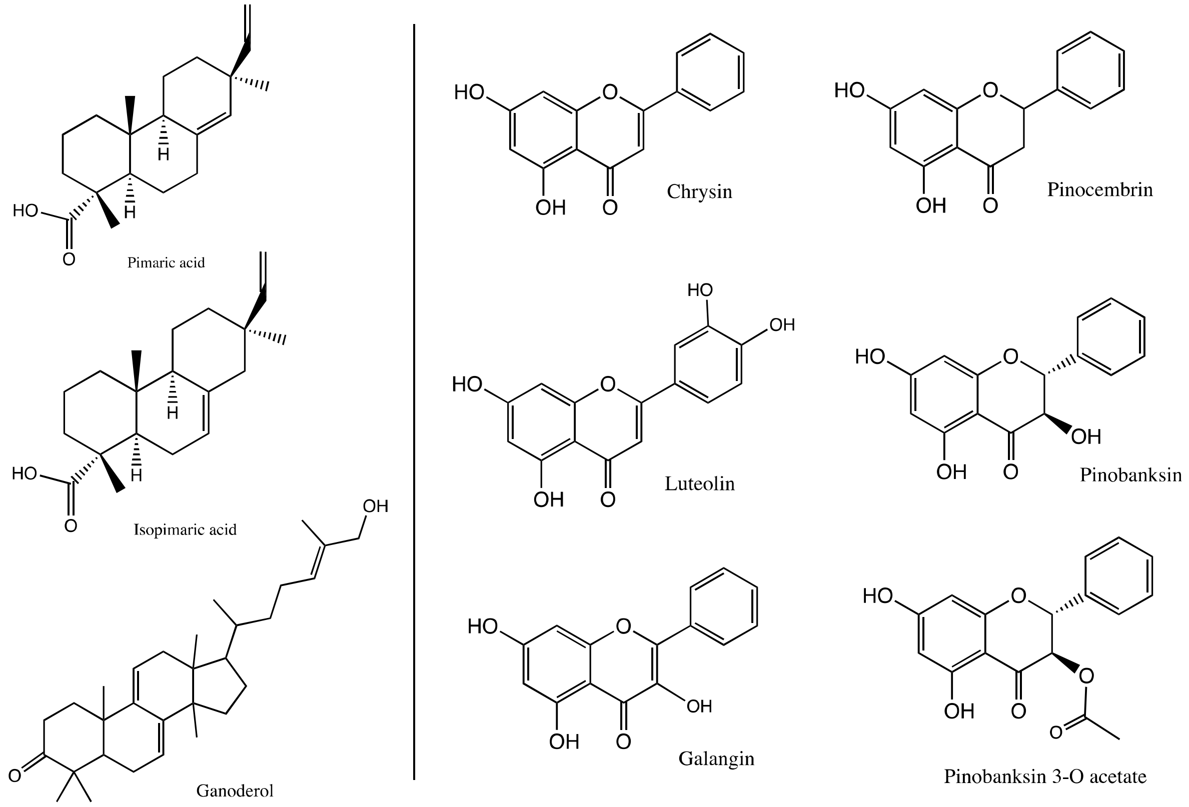

Overexpressed in Greek samples *Overexpressed in Chinese samples*

Figure S8: Structures of key compounds responsible for the differentiation (VIPs) of Greek and Chinese propolis samples in positive and negative ion mode according to Table 1.

Table S1: List of the standard compounds' mixture injected for dereplication purposes in negative ion mode (exact mass and molecular formula of m/z ion). Compounds are shorted according to their elution order with noted their spectrometric and chromatographic features.

No	Rt*	Compound	[M-H]$^-$	Formula [M-H]$^-$
**1**	3.30	Protocatechuic acid	153	$C_7H_5O_4$
**2**	3.93	Adipic acid	145	$C_6H_9O_4$
**3**	4.56	Chlorogenic acid	353	$C_{16}H_{17}O_9$
**4**	5.92	Caffeic acid	179.0350	$C_9H_7O_4$
**5**	7.20	Vanillin	151	$C_8H_7O_3$
**6**	7.84	Orientin	447	$C_{21}H_{19}O_{11}$
**7**	7.98	p-Coumaric acid	163.0399	$C_9H_7O_3$
**9**	8.49	Isoferulic acid	193.0508	$C_{10}H_9O_4$
**10**	8.59	Vitexin	431	$C_{21}H_{19}O_{10}$
**11**	9.24	Suberic acid	173	$C_8H_{13}O_4$
12	9.67	*t*-Resveratrol	227	$C_{14}H_{11}O_3$
**13**	9.73	Naringin	579	$C_{27}H_{31}O_{14}$
**14**	9.95	Hesperidin	609	$C_{28}H_{33}O_{15}$
**8**	10.19	Ferulic acid	193.0506	$C_{10}H_9O_4$
**15**	10.22	Phloridzin	435	$C_{21}H_{23}O_{10}$
**16**	10.24	Diosmin	607	$C_{28}H_{31}O_{15}$
**17**	10.25	Protocatechuic acid ethyl ester	181	$C_9H_9O_4$
**18**	10.93	Eriodictyol	287	$C_{15}H_{11}O_6$
**19**	11.36	Daidzein	253.0976	$C_{15}H_9O_4$
**21**	12.04	Kaempferol	285.0406	$C_{15}H_9O_6$
**22**	12.04	Luteolin	285.0834	$C_{15}H_9O_6$
**53**	12.05	Quercetin	301.0354	$C_{15}H_9O_7$
**24**	12.12	Naringenin	271	$C_{15}H_{11}O_5$
**23**	12.69	Pinobanksin	271.0610	$C_{15}H_{11}O_5$
**30**	12.82	Genistein	269.0453	$C_{15}H_9O_5$
**31**	13.45	Hesperetin	301	$C_{16}H_{13}O_6$
**32**	13.11	Kaempferide	299	$C_{16}H_{11}O_6$
**48**	13.48	Isorhamnetin	315.0511	$C_{16}H_{11}O_7$
**28**	14.51	Apigenin	269.0453	$C_{16}H_{13}O_4$
**35**	14.53	Sakuranetin	285	$C_{16}H_{13}O_5$
**36**	14.53	Isosakuranetin	285	$C_{16}H_{13}O_5$
**37**	14.67	Pinocembrin	255.0662	$C_{15}H_{11}O_4$
**25**	14.69	Rhamnetin	315.0524	$C_{16}H_{11}O_7$
**38**	15.05	Pinobanksin 3-*O*-acetate	313.0720	$C_{17}H_{13}O_6$
**39**	15.22	CAPE	283.0976	$C_{17}H_{15}O_4$
**20**	15.21	Chrysin	253.0507	$C_{15}H_9O_4$
**33**	15.50	Diosmetin	299	$C_{16}H_{11}O_6$
**29**	15.69	Galangin	269.0455	$C_{15}H_9O_5$
**40**	15.80	Acacetin	283	$C_{16}H_{11}O_5$
**41**	16.08	Rosmarinic acid	359	$C_{18}H_{15}O_8$
**26**	17.02	PIN-7ME	269.0819	$C_{16}H_{13}O_4$
**27**	17.25	Pinostrobin	269.0817	$C_{16}H_{13}O_4$
**34**	18.80	Chrysoeriol	299	$C_{16}H_{11}O_6$

**44**	24.08	Corosolic acid	471	$C_{30}H_{47}O_4$
**42**	24.28	Maslinic acid	471	$C_{30}H_{47}O_4$
**43**	28.09	Ursolic acid	455	$C_{30}H_{47}O_3$

© 2020 by the authors. Submitted for possible open access publication under the terms and conditions of the Creative Commons Attribution (CC BY) license (http://creativecommons.org/licenses/by/4.0/).
